# Lactate metabolic plasticity across the menopausal transition: a hypothesis-generating framework linking ovarian hormone variability, exercise, and skeletal muscle remodeling

**DOI:** 10.3389/fphys.2026.1954454

**Published:** 2026-10-28

**Authors:** Xichen Duan, Dong Du

**Affiliations:** 1 Jilin Sport University, Changchun, Jilin, China; 2 Guangxi Sports College, Nanning, China

**Keywords:** exercise training, lactate kinetics, lactate metabolism, menopausal transition, metabolic flexibility, perimenopause, skeletal muscle

## Abstract

The menopausal transition combines marked ovarian-hormone variability with changes in body composition, insulin sensitivity, vascular function, skeletal muscle, and exercise substrate use. Established lactate-shuttle, maximal-lactate-steady-state, metabolic-flexibility, and Lactate-Carbohydrate models already explain that lactate is an oxidative fuel, a gluconeogenic precursor, and an informative but protocol-dependent marker of substrate use rather than a waste product. The unresolved menopause-specific question is whether changing ovarian status alters the coordination of systemic lactate appearance and disappearance during exercise and recovery. This Mini Review therefore distinguishes evidence from natural perimenopause from evidence in established postmenopause and other populations, defines testable stage-specific hypotheses, and proposes a component measurement profile for future studies. The profile combines standardized absolute and relative exercise workloads, serial blood lactate concentrations, stable-isotope estimates of lactate rate of appearance, rate of disappearance, oxidation and recycling, repeated estradiol and follicle-stimulating hormone measurements, and muscle or vascular phenotyping. A structured narrative search identified no study that directly measured lactate flux or whole-body metabolic clearance during natural perimenopause. The novelty of the proposed framework therefore lies in its menopause-specific evidence hierarchy and study design, not in reclassifying lactate biology. Longitudinal studies are needed before any component can be treated as a menopausal biomarker or used to individualize exercise prescription.

## 1 Introduction

The transition into menopause is a biological process spanning several years, characterized by alterations in menstrual cycle regularity and progression toward the final menstrual period, rather than being defined by a single estradiol concentration or age (Harlow et al., 2012). Estradiol can fluctuate substantially during perimenopause before becoming persistently lower after menopause, whereas follicle-stimulating hormone (FSH) generally rises as ovarian reserve declines (Harlow et al., 2012). Therefore, perimenopause should not be regarded as a uniform state of estrogen deficiency or indiscriminately grouped with established postmenopause (Harlow et al., 2012).

Direct longitudinal human evidence links the menopausal transition with increases in abdominal and visceral fat, loss of lean or muscle mass, lower energy expenditure, and a less favorable cardiometabolic profile (Janssen et al., 2008; Lovejoy et al., 2008; Greendale et al., 2019; El Khoudary et al., 2020; Juppi et al., 2020). Quantitative findings illustrate the scale and heterogeneity of these changes. In one four-year cohort, sleeping energy expenditure fell by 7.9% in women who became postmenopausal versus 5.3% in premenopausal controls (Lovejoy et al., 2008). In a controlled cross-sectional exercise study of 41 women (19 premenopausal, 8 perimenopausal, and 14 postmenopausal), postmenopausal participants had 33% lower whole-body fat oxidation, 19% lower energy expenditure, and 4.28 kg less lean body mass than premenopausal participants; adjustment for lean mass reduced the fat-oxidation difference to 23% and eliminated the energy-expenditure difference (Abildgaard et al., 2013). These comparisons do not establish a lactate mechanism, but they identify active muscle mass and substrate use as necessary covariates in menopause-stage studies.

Most menopause research has focused on adiposity, glucose regulation, lipid oxidation, vascular function, muscle mass, and cardiorespiratory fitness (Janssen et al., 2008; Lovejoy et al., 2008; Abildgaard et al., 2013; Greendale et al., 2019; El Khoudary et al., 2020; Juppi et al., 2020; Kleis-Olsen et al., 2024). Lactate physiology itself is not a new field: the rejection of lactate as merely a waste product, the lactate-shuttle framework, maximal lactate steady state (MLSS), and exercise-based metabolic-flexibility models have been developed and debated across decades (Brooks, 1986; Beneke, 2003; Gladden, 2004; Faude et al., 2009; Goodpaster and Sparks, 2017; Hui et al., 2017; Brooks, 2018; Ferguson et al., 2018; San-Millán and Brooks, 2018; Rabinowitz and Enerbäck, 2020; Alkhatib, 2022). The Lactate-Carbohydrate Model further relates blood lactate concentration to the balance between carbohydrate and fat use during graded exercise (Alkhatib, 2022). What remains missing is a reproductive-stage analysis that tests lactate flux during natural perimenopause. The cited natural-perimenopause literature addresses staging, body composition, lean mass, and endothelial function (Harlow et al., 2012; Moreau et al., 2012; Greendale et al., 2019; Juppi et al., 2020), whereas the available postmenopausal study of MLSS measured concentration-based exercise thresholds rather than lactate rate of appearance (Ra), rate of disappearance (Rd), oxidation, recycling, or whole-body metabolic clearance (Gil-Rey et al., 2019). Ra denotes the rate of lactate appearance in the systemic circulation, whereas Rd denotes the rate of lactate disappearance from the systemic circulation; these are separate tracer-derived fluxes and are not interchangeable.

The menopause-specific aim of this Mini Review is to define how natural reproductive stage and ovarian-hormone variability could be tested as determinants of lactate appearance, transport, oxidation, recycling, and disappearance during exercise and recovery. We do not propose a new general theory of lactate metabolism. Instead, we extend established lactate-shuttle, MLSS, and metabolic-flexibility concepts into an evidence-stratified framework that separates direct natural-perimenopause evidence from postmenopausal and general exercise evidence (Brooks, 1986; Beneke, 2003; Gladden, 2004; Faude et al., 2009; Goodpaster and Sparks, 2017; Hui et al., 2017; Brooks, 2018; Ferguson et al., 2018; San-Millán and Brooks, 2018; Gil-Rey et al., 2019; Rabinowitz and Enerbäck, 2020; Alkhatib, 2022). The primary hypothesis is not that perimenopausal women consistently have high or low blood lactate; it is that reproductive stage and within-person hormone variability may alter the coordination or variability of component outcomes. The conceptual model is termed the lactate metabolic plasticity framework, and its observable variables are termed component outcomes or a component measurement profile. **Figure 1** summarizes the conceptual framework, distinguishing established lactate physiology from menopause-related evidence and untested hypotheses.

**Figure 1:**
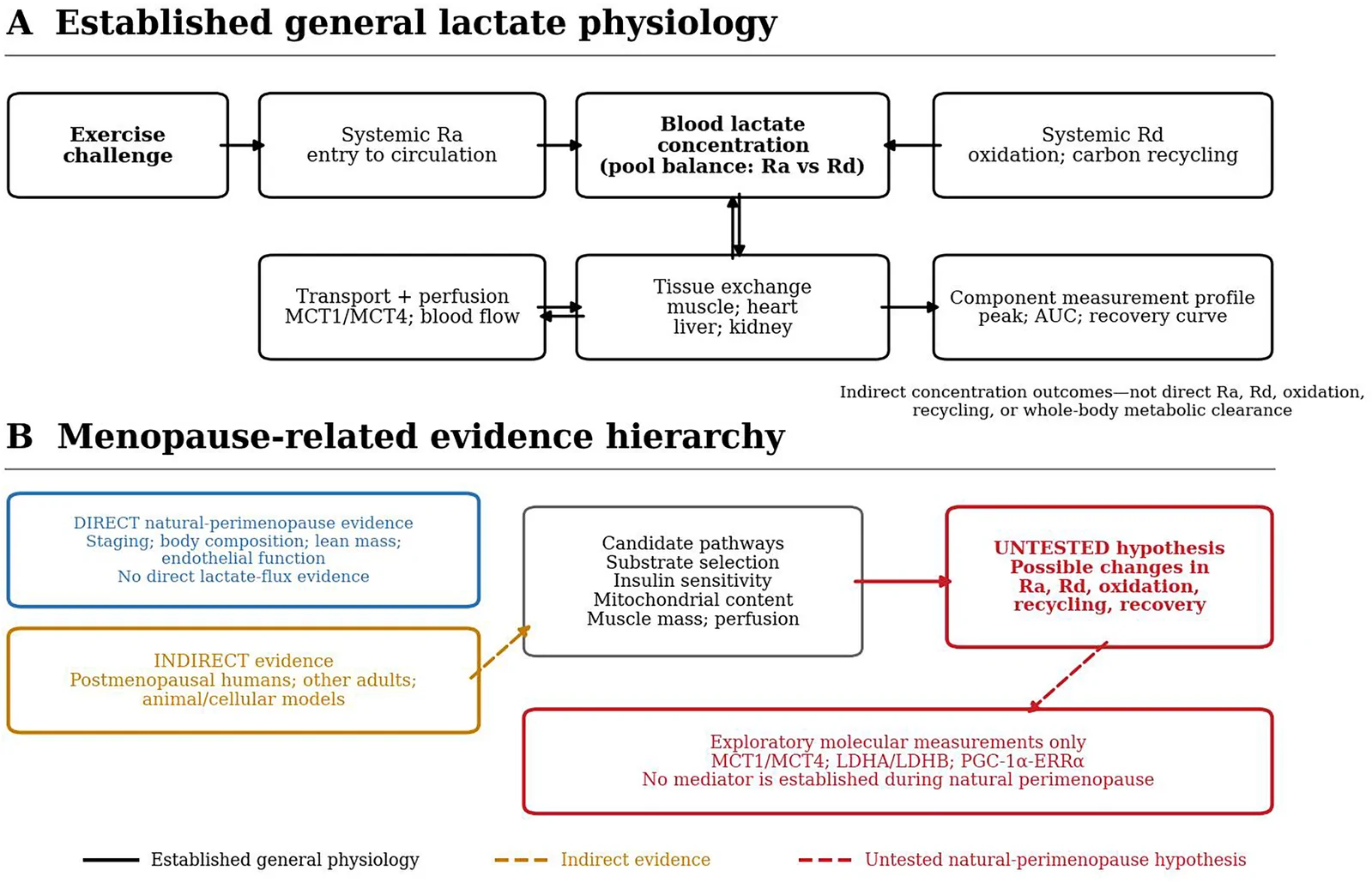
Evidence-stratified, hypothesis-generating model of the lactate metabolic plasticity framework across the menopausal transition. Panel **(A)** (black solid arrows) shows established general relationships: exercise changes glycolytic lactate production and systemic Ra; blood concentration reflects the balance between Ra and Rd; and MCT1/MCT4, perfusion, oxidation, and gluconeogenesis contribute to tissue exchange and disappearance (Brooks, 1986; Pilegaard et al., 1999; Dubouchaud et al., 2000; Gladden, 2004; Juel et al., 2004; Thomas et al., 2005; Hashimoto and Brooks, 2008; Summermatter et al., 2013; Goodpaster and Sparks, 2017; Hui et al., 2017; Brooks, 2018; Ferguson et al., 2018; Rabinowitz and Enerbäck, 2020; Benítez-Muñoz et al., 2024). Peak concentration, area under the curve, and the concentration-recovery curve are indirect, protocol-dependent component outcomes and do not equal production, Rd, oxidation, recycling, or whole-body metabolic clearance (Gladden, 2004; Thomas et al., 2005; Maciejewski et al., 2016; Hui et al., 2017; Brooks, 2018; Ferguson et al., 2018; Rabinowitz and Enerbäck, 2020). Panel **(B)** separates menopause-related evidence. Direct natural-perimenopause human evidence (blue) supports staging and changes in body composition, lean mass, or endothelial function (Harlow et al., 2012; Moreau et al., 2012; Greendale et al., 2019; Juppi et al., 2020), but no direct lactate-flux measurement was identified under the eligibility criteria in **Supplementary Methods S1**. Indirect evidence (amber dashed arrows) comes from postmenopausal humans, experimental models, and other exercise populations (Pilegaard et al., 1999; Dubouchaud et al., 2000; Juel et al., 2004; Thomas et al., 2005; Bishop et al., 2008; Moreau et al., 2012; Abildgaard et al., 2013; Moreau et al., 2013; Summermatter et al., 2013; Cavalcanti-de-Albuquerque et al., 2014; Moreau and Hildreth, 2014; Gupte et al., 2015; Nyberg et al., 2017; Mandrup et al., 2018; Torres et al., 2018; Gil-Rey et al., 2019; Pellegrino et al., 2022; Yoh et al., 2023; Benítez-Muñoz et al., 2024; Kleis-Olsen et al., 2024; Mølmen et al., 2025; Monferrer-Marín et al., 2026). Red dashed arrows denote the untested hypothesis that ovarian-hormone variability and correlated changes in substrate selection, insulin sensitivity, mitochondrial content, muscle mass, or perfusion alter integrated lactate kinetics. Effects on MCT1, MCT4, LDHA, LDHB, or PGC-1α–ERRα signaling during natural perimenopause remain untested and are not established mediators (Pilegaard et al., 1999; Dubouchaud et al., 2000; Juel et al., 2004; Thomas et al., 2005; Summermatter et al., 2013; Yoh et al., 2023; Benítez-Muñoz et al., 2024).

### 1.1 Review approach and evidence hierarchy

PubMed/MEDLINE and Europe PMC were searched from database inception through 30 August 2026. The complete core strings, supplementary search families, and platform-specific field and date-expansion rules are reported in **Supplementary Methods S1**. The core search combined menopausal-stage terms with lactate concentration, kinetics, turnover, rate of appearance (Ra), rate of disappearance (Rd), oxidation, whole-body metabolic clearance, MCT1/MCT4, and LDHA/LDHB terms, together with exercise, training, or skeletal-muscle terms. Supplementary searches paired estradiol, estrogen, ovarian-hormone, and exogenous-hormone terms with perfusion and endothelial function; mitochondrial function, respiration, substrate oxidation, and insulin sensitivity; PGC-1α, ERRα, LDH, and monocarboxylate transporters; or histone lactylation. Crossref and publisher or PubMed records were used only to verify bibliographic metadata and DOI information. Reference lists and available forward-citation links for key reviews and original studies were screened for additional records.

One reviewer screened titles and abstracts and retrieved full texts when a record involved a relevant reproductive stage, lactate concentration or flux, exercise or skeletal muscle, or a mechanism prespecified in the evidence hierarchy. Direct natural-perimenopause eligibility required all of the following: a human cohort undergoing the natural menopausal transition; an identifiable reproductive stage based on menstrual history, STRAW + 10-compatible criteria, or an equivalent reported definition; and direct measurement of lactate Ra, Rd, oxidation, carbon recycling, or model-derived whole-body metabolic clearance at rest, during exercise, or during standardized recovery. Concentration-only studies were retained as indirect lactate evidence but were not classified as direct flux evidence.

Records were excluded from the direct natural-perimenopause category when they involved only established postmenopause, surgical or treatment-induced menopause, an unidentifiable reproductive stage, no eligible lactate outcome, or a disease-specific cohort in which disease and menopause effects could not be separated. Staging guidance and systematic or narrative reviews were retained for definitions and citation chaining; postmenopausal human studies, other adult studies, animal models, and cell studies were retained only as indirect evidence or biological plausibility. Screening, evidence classification, and extraction were performed by one reviewer rather than in duplicate. Applying these criteria identified no eligible direct natural-perimenopause flux study. This statement is therefore bounded by the documented search and eligibility rules and is not a claim that absence of evidence proves biological absence.

## 2 Established lactate models and the menopause-specific extension

Lactate is constantly produced through the reversible lactate dehydrogenase reaction, including in adequately oxygenated tissues, and its roles as an oxidative fuel, gluconeogenic precursor, and circulating carbon carrier are established rather than novel claims of this Review (Brooks, 1986; Gladden, 2004; Hui et al., 2017; Brooks, 2018; Ferguson et al., 2018; Rabinowitz and Enerbäck, 2020). The lactate-shuttle framework describes exchange among producing and consuming cells and organs (Brooks, 1986; Brooks, 2018). MLSS identifies the highest constant workload at which blood lactate does not continually accumulate, but its measured concentration is protocol dependent; Beneke reported values of 5.1, 4.9, and 4.3 mmol·L^−1^ in the same study population when three methodological definitions were applied (Beneke, 2003). **Table 1** summarizes established lactate concepts and distinguishes them from the proposed menopause-specific extension. Accordingly, neither an exercise-induced concentration increase nor a fixed concentration threshold directly identifies tissue hypoxia, production, oxidation, or a distinct anaerobic state (Beneke, 2003; Gladden, 2004; Faude et al., 2009; Brooks, 2018; Ferguson et al., 2018; Rabinowitz and Enerbäck, 2020).

**Table 1:** Established lactate concepts and the menopause-specific extension.

Concept	Established contribution	Evidence base	Role in this Review
Lactate shuttle	Explains cell-to-cell and organ-to-organ lactate exchange, oxidation, and gluconeogenic recycling (Brooks, 1986; Brooks, 2018).	General and exercise physiology	Provides the mechanistic foundation; it is not renamed or claimed as novel.
MLSS and threshold models	Characterize sustainable exercise intensity and the balance reflected in blood lactate, with protocol-dependent values (Beneke, 2003; Faude et al., 2009).	Athletic and general populations; concentration-based postmenopausal testing (Gil-Rey et al., 2019)	Support standardized exercise challenges but do not measure Ra, Rd, oxidation, or recycling.
Exercise metabolic flexibility	Relates workload-dependent lactate to fat and carbohydrate oxidation (San-Millán and Brooks, 2018).	Professional athletes, less-fit adults, and metabolic-syndrome populations	Supports paired lactate and substrate-oxidation measurements; reproductive-stage effects remain untested.
Lactate-Carbohydrate Model	Models relative carbohydrate use as a function of blood lactate concentration (Alkhatib, 2022).	Twenty-one healthy men	Provides quantitative precedent but requires validation in women and across menopausal stages.
Menopause-specific lactate metabolic plasticity framework	Integrates reproductive stage, repeated ovarian hormones, standardized exercise and recovery, direct lactate flux, and muscle or vascular phenotyping.	Hypothesis-generating; no direct natural-perimenopause flux study identified	Defines the novel application and the measurements required to test it.

The first four rows summarize established concepts. The final row identifies the proposed menopause-specific extension and its current evidence boundary.

Existing models address complementary levels of lactate physiology. The lactate shuttle describes intercellular and interorgan carbon transfer (Brooks, 1986; Brooks, 2018); MLSS and threshold concepts characterize sustainable exercise intensity but remain method dependent (Beneke, 2003; Faude et al., 2009); the exercise metabolic-flexibility approach compares blood lactate with fat and carbohydrate oxidation across workloads (San-Millán and Brooks, 2018); and the Lactate-Carbohydrate Model formalizes the relation between blood lactate concentration and relative carbohydrate use (Alkhatib, 2022). In the latter study of 21 healthy men, blood lactate explained 86% of the variability in relative carbohydrate use, while the individualized model constant explained 55% of maximal fat oxidation variability and 44% of crossover-point variability (Alkhatib, 2022). These results establish methodological precedent, but their male sample and cross-sectional design do not answer whether ovarian-hormone variability modifies lactate flux in perimenopausal women.

The proposed lactate metabolic plasticity framework is therefore a menopause-specific research extension, not a replacement for those models. Its distinctive contribution is to integrate STRAW + 10 reproductive staging, repeated ovarian-hormone measurements, exercise and recovery, and direct tracer outcomes within one evidence hierarchy that explicitly separates natural perimenopause from established postmenopause and other populations (Brooks, 1986; Beneke, 2003; Gladden, 2004; Faude et al., 2009; Harlow et al., 2012; Goodpaster and Sparks, 2017; Hui et al., 2017; Brooks, 2018; Ferguson et al., 2018; San-Millán and Brooks, 2018; Gil-Rey et al., 2019; Rabinowitz and Enerbäck, 2020; Alkhatib, 2022). The framework encompasses systemic appearance, proton-coupled transport, oxidative or gluconeogenic disposal and carbon recycling, and the post-exercise concentration response. It is not a validated biomarker, composite score, physiological index, or phenotype, and no cutoff or favorable direction has been established. Its component measurement profile comprises external power and oxygen uptake; serial blood lactate concentration; peak and time to peak; a prespecified area under the curve; and a modeled concentration-recovery response. Tracer-derived Ra, Rd, oxidation, recycling, and whole-body metabolic clearance must be reported separately. A low peak concentration cannot be interpreted as beneficial in isolation because it may reflect efficient oxidation, low glycolytic flux, low exercise intensity, reduced active muscle mass, or an insufficient physiological challenge (Gladden, 2004; Moreau et al., 2013; Brooks, 2018; Ferguson et al., 2018; Rabinowitz and Enerbäck, 2020). **Table 2** summarizes established measurement methods, proposed applications, and essential controls for future studies of this framework.

**Table 2:** Established methods and authors’ proposed applications for testing the lactate metabolic plasticity framework across the menopausal transition.

Domain and role	Recommended measurement or outcome	Current evidence and feasibility	Essential controls
Reproductive status Established staging Proposed user policy	For participants not using exogenous hormones: STRAW + 10 stage, menstrual history, repeated estradiol and FSH, cycle timing, and assay details (Harlow et al., 2012). For hormone users: prespecified separate-stratum or exclusion rule for primary natural-stage analyses.	STRAW+10 is established staging guidance. Separate handling of participants whose staging criteria are obscured is the authors’ proposed policy; no direct perimenopausal lactate validation exists.	Do not assign natural stage from contemporaneous hormones during exogenous use. Use documented pre-treatment menstrual history when valid; otherwise exclude from primary stage comparison or analyze separately. Record formulation, route, dose, duration, adherence, indication, and timing.
Exercise challenge Established method Proposed application	Paired fixed absolute-workload and relative-domain stages; power, duration, VO_2_peak, thresholds, and completed work (Faude et al., 2009; Moreau et al., 2013; San-Millán and Brooks, 2018)	General exercise physiology; no direct perimenopausal validation; high feasibility.	Familiarization, active muscle mass, fitness, device calibration, and identical stage duration
Blood lactate concentration Established measure Proposed outcome set	Rest, each exercise stage, immediate post-exercise, delayed peak, and serial standardized recovery; peak, time to peak, AUC, and fitted curve reported separately (Thomas et al., 2005; Faude et al., 2009; Maciejewski et al., 2016; San-Millán and Brooks, 2018; Gil-Rey et al., 2019)	No direct natural-perimenopause study; concentration is indirect. High feasibility.	Sampling site, analyzer and analytical CV, temperature, posture, and active versus passive recovery
Lactate flux Established tracer method Proposed application	Stable-isotope Ra, Rd, oxidation, recycling, and model-derived whole-body metabolic clearance (Gladden, 2004; Hui et al., 2017; Brooks, 2018; Ferguson et al., 2018; Hui et al., 2020; Rabinowitz and Enerbäck, 2020)	No direct natural-perimenopause study; specialized expertise and moderate-to-low feasibility.	Tracer equilibration or non-steady-state model, diet, fasting, product measurements, and model assumptions
Muscle mechanism Proposed exploratory	MCT1, MCT4, LDHA, LDHB, PGC-1α, ERRα, ERα, mitochondrial respiration, fiber type, and capillary density (Pilegaard et al., 1999; Dubouchaud et al., 2000; Juel et al., 2004; Thomas et al., 2005; Summermatter et al., 2013; Yoh et al., 2023; Benítez-Muñoz et al., 2024)	No direct human perimenopause evidence; low-to-moderate feasibility because biopsy and laboratory resources are required.	Fiber type, biopsy timing, training status, tissue handling, and blinded assay procedures
Perfusion Established vascular method Proposed link	Exercising-limb blood flow, oxygen delivery, endothelial function, and capillary density (Moreau et al., 2012; Moreau et al., 2013; Moreau and Hildreth, 2014; Nyberg et al., 2017; Mandrup et al., 2018; Mølmen et al., 2025)	Direct reproductive-stage endothelial evidence, but the perfusion–lactate link is untested; moderate feasibility.	Blood pressure, vascular risk, medication, ambient temperature, and concurrent workload
Training response Proposed outcome	Change in power–lactate relationship, serial recovery concentration, and tracer flux after intervention (Pilegaard et al., 1999; Dubouchaud et al., 2000; Juel et al., 2004; Thomas et al., 2005; Bishop et al., 2008; Faude et al., 2009; Summermatter et al., 2013; Nyberg et al., 2017; Mandrup et al., 2018; San-Millán and Brooks, 2018; Gil-Rey et al., 2019; Benítez-Muñoz et al., 2024; Mølmen et al., 2025; Monferrer-Marín et al., 2026)	Postmenopausal and other-adult evidence only; no natural-perimenopause flux trial; moderate feasibility.	Matched training dose, adherence, energy balance, baseline fitness, and repeated-test reliability
Clinical relevance Established measures Proposed covariates	Insulin sensitivity, total and visceral adiposity, muscle mass and function, physical activity, and cardiorespiratory fitness (Janssen et al., 2008; Lovejoy et al., 2008; Abildgaard et al., 2013; Nyberg et al., 2017; Mandrup et al., 2018; Greendale et al., 2019; El Khoudary et al., 2020; Juppi et al., 2020; Kleis-Olsen et al., 2024; Mølmen et al., 2025)	Evidence varies by variable; high-to-moderate feasibility. These are not proof of lactate-mediated symptoms.	Age, MHT, medications, smoking, disease, and prespecified confounder versus mediator roles
Reliability and analysis Established principles Proposed application	Duplicate baseline tests; analytical and within-participant CV, ICC, typical error, smallest detectable change; mixed-effects or justified kinetic model	No perimenopause-specific reliability data; high feasibility for duplicate concentration testing.	Prespecified sampling window, participant random effects, stage×time/intensity interaction, multiplicity, missing data, and sensitivity analysis
Histone lactylation Proposed exploratory	Tissue-specific lactylation assays, only within a separately powered mechanistic substudy (Zhang et al., 2019)	No menopausal exercise evidence; low feasibility; not part of the core component measurement profile.	Tissue, cell composition, biopsy timing, assay validation, and no inference from blood lactate alone

‘Established method’ denotes staging, exercise-testing, lactate-assay, tracer, vascular, reliability, or analytic methods supported outside this specific application. ‘Authors’ proposed application,’ ‘proposed outcome set,’ and ‘proposed mechanism’ denote recommendations for future perimenopause studies that have not been directly validated in natural perimenopause. These labels separate methodological precedent from the unvalidated lactate metabolic plasticity framework.

Monocarboxylate transporter 1 (MCT1) and 4 (MCT4) are bidirectional monocarboxylate/proton cotransporters, with net transport direction determined by substrate and proton gradients (Pilegaard et al., 1999; Dubouchaud et al., 2000; Juel et al., 2004; Thomas et al., 2005; Benítez-Muñoz et al., 2024). MCT1 is commonly associated with oxidative muscle phenotypes and lactate uptake, whereas MCT4 is enriched in more glycolytic fibers and supports lactate export during high glycolytic flux (Pilegaard et al., 1999; Dubouchaud et al., 2000; Juel et al., 2004; Thomas et al., 2005; Benítez-Muñoz et al., 2024). Skeletal muscle MCT content can increase after endurance or high-intensity training, but responses vary by fiber type, exercise mode, sampling time, and training status (Pilegaard et al., 1999; Dubouchaud et al., 2000; Juel et al., 2004; Thomas et al., 2005; Benítez-Muñoz et al., 2024). Women were markedly underrepresented—approximately 10% of human participants—in a recent systematic review of exercise-induced MCT responses (Benítez-Muñoz et al., 2024). These are general physiological and training observations, not direct evidence from natural perimenopause.

Peroxisome proliferator-activated receptor-γ coactivator 1α (PGC-1α) provides a plausible molecular connection between exercise-induced mitochondrial remodeling and lactate oxidation (Summermatter et al., 2013). In experimental muscle models, PGC-1α increased LDHB and reduced LDHA expression through estrogen-related receptor α (ERRα), shifting the LDH profile toward lactate oxidation and altering whole-body lactate homeostasis (Summermatter et al., 2013). ERRα is an orphan nuclear receptor and must not be conflated with classical estrogen receptor α (ERα) (Summermatter et al., 2013; Yoh et al., 2023). No human study has shown that fluctuating estradiol regulates the PGC-1α–ERRα–LDHB axis during natural perimenopause (Summermatter et al., 2013; Yoh et al., 2023).

## 3 Why the menopausal transition could perturb lactate handling

Evidence boundary. Sections 3.1 and 3.2 summarize biological plausibility from established postmenopause, other human populations, and experimental models. Unless explicitly labeled as direct natural-perimenopause evidence, mitochondrial function, perfusion, MCT1/MCT4, LDHA/LDHB, and PGC-1α–ERRα signaling are candidate pathways only; none is an established mediator of altered lactate metabolism during the natural menopausal transition.

### 3.1 Substrate selection and mitochondrial capacity

In experimental animal and cellular models, estradiol and its receptors influence insulin signaling, substrate oxidation, mitochondrial membrane characteristics, redox balance, and mitochondrial gene expression (Cavalcanti-de-Albuquerque et al., 2014; Gupte et al., 2015; Torres et al., 2018; Pellegrino et al., 2022; Yoh et al., 2023). In rodents, ovariectomy may reduce skeletal-muscle mitochondrial respiration, while estradiol replacement can recover aspects of respiratory function and redox balance (Cavalcanti-de-Albuquerque et al., 2014; Torres et al., 2018). These findings provide biological plausibility only and cannot be equated with natural perimenopause, which combines fluctuating ovarian function with aging, behavioral changes, and altered body composition rather than abrupt surgical hormone withdrawal (Janssen et al., 2008; Lovejoy et al., 2008; Harlow et al., 2012; Greendale et al., 2019; Juppi et al., 2020).

Human evidence remains indirect but provides quantitative constraints for the proposed pathway. In the 41-participant exercise study described above, postmenopausal women showed 33% lower whole-body fat oxidation and 4.28 kg lower lean body mass than premenopausal women, while adjustment for lean mass reduced the fat-oxidation difference to 23% (Abildgaard et al., 2013). In a separate cross-sectional study of 22 postmenopausal women, the 11 MHT users had higher absolute mitochondrial respiratory capacity and enzyme markers of mitochondrial content than 11 matched non-users, but group differences were absent after respiration was normalized to citrate synthase activity (Kleis-Olsen et al., 2024). Neither study measured lactate flux. Together they argue against attributing a concentration difference to intrinsic mitochondrial dysfunction without accounting for active muscle mass, mitochondrial quantity, exercise workload, and hormone exposure.

Reduced mitochondrial content or pyruvate oxidation could theoretically increase systemic lactate appearance when glycolytic flux exceeds oxidative disposal (Gladden, 2004; Abildgaard et al., 2013; Goodpaster and Sparks, 2017; Brooks, 2018; Kleis-Olsen et al., 2024). An alternative concentration pattern would be an unchanged peak followed by a slower decline; however, such a concentration-recovery curve is jointly influenced by distribution, ongoing production, perfusion, oxidation, gluconeogenesis, and recovery activity and cannot directly demonstrate lower Rd or lower whole-body metabolic clearance (Pilegaard et al., 1999; Dubouchaud et al., 2000; Gladden, 2004; Thomas et al., 2005; Brooks, 2018; Ferguson et al., 2018; Rabinowitz and Enerbäck, 2020). Reduced exercise capacity or active muscle mass could also lower total lactate production despite impaired cellular metabolism (Gladden, 2004; Brooks, 2018; Ferguson et al., 2018; Juppi et al., 2020; Rabinowitz and Enerbäck, 2020). These alternatives are testable hypotheses, not observed perimenopausal patterns. Increased variability is hypothesized because the menopausal transition combines marked within-person fluctuations in ovarian hormones with heterogeneous timing and magnitude of changes in menstrual cyclicity, body composition, insulin sensitivity, symptoms, fitness, and vascular function (Janssen et al., 2008; Lovejoy et al., 2008; Harlow et al., 2012; Abildgaard et al., 2013; Greendale et al., 2019; El Khoudary et al., 2020; Juppi et al., 2020; Kleis-Olsen et al., 2024). Hormone-sensitive processes may also respond over different time scales, so the same participant could show different component outcomes across visits, while differing hormone trajectories and metabolic backgrounds could widen between-person distributions within the same STRAW + 10 stage. This variability hypothesis remains untested and requires repeated within-person exercise challenges and hormone measurements; it must not be interpreted as evidence that perimenopause causes variable or uniformly elevated lactate.

### 3.2 Transport, perfusion, and hormone-sensitive regulation

Lactate exchange is influenced by tissue blood flow, capillary surface area, transmembrane transport, intracellular LDH balance, and mitochondrial oxidative capacity (Pilegaard et al., 1999; Dubouchaud et al., 2000; Juel et al., 2004; Thomas et al., 2005; Brooks, 2018; Benítez-Muñoz et al., 2024). Direct reproductive-stage evidence shows a graded decline in brachial-artery flow-mediated dilation across 132 healthy women: 9.9 ± 2.1% before menopause, 8.2 ± 2.5% in early perimenopause, 6.5 ± 1.9% in late perimenopause, 5.5 ± 1.9% in early postmenopause, and 4.7 ± 1.7% in late postmenopause (Moreau et al., 2012). This vascular gradient makes perfusion a plausible covariate or mediator, but the study did not measure exercising-muscle blood flow or lactate flux. The proposed perfusion-to-lactate pathway therefore remains untested (Moreau et al., 2012; Moreau and Hildreth, 2014).

Estrogen status may modify some vascular adaptations to exercise (Moreau et al., 2012; Moreau et al., 2013; Moreau and Hildreth, 2014; Pellegrino et al., 2022). In a randomized study of postmenopausal women, endurance training improved endothelial function in the estradiol-treated group but not placebo, a result consistent with—but not proof of—a permissive estrogen effect on that vascular outcome (Moreau et al., 2013). Other studies in early postmenopausal women report vascular, mitochondrial, insulin-sensitivity, and muscle adaptations to high-intensity training, showing that exercise responsiveness is not globally abolished after menopause (Nyberg et al., 2017; Mandrup et al., 2018). These findings can coexist because estrogen may modify selected pathways while exercise activates multiple estrogen-dependent and estrogen-independent mechanisms (Moreau et al., 2012; Moreau et al., 2013; Cavalcanti-de-Albuquerque et al., 2014; Moreau and Hildreth, 2014; Gupte et al., 2015; Nyberg et al., 2017; Mandrup et al., 2018; Torres et al., 2018; Pellegrino et al., 2022; Yoh et al., 2023).

No human study has established that natural perimenopause changes skeletal muscle MCT1, MCT4, LDHA, LDHB, or PGC-1α–ERRα signaling (Pilegaard et al., 1999; Dubouchaud et al., 2000; Juel et al., 2004; Thomas et al., 2005; Summermatter et al., 2013; Yoh et al., 2023; Benítez-Muñoz et al., 2024). These proteins are candidate mechanisms, not established mediators of menopausal changes in lactate metabolism (Pilegaard et al., 1999; Dubouchaud et al., 2000; Juel et al., 2004; Thomas et al., 2005; Summermatter et al., 2013; Yoh et al., 2023; Benítez-Muñoz et al., 2024). Age, total and visceral adiposity, active muscle mass, habitual physical activity, cardiorespiratory fitness, insulin sensitivity, medication use, and MHT may confound stage comparisons, modify associations, or lie on the causal pathway (Janssen et al., 2008; Lovejoy et al., 2008; Moreau et al., 2012; Abildgaard et al., 2013; Moreau et al., 2013; Cavalcanti-de-Albuquerque et al., 2014; Moreau and Hildreth, 2014; Gupte et al., 2015; Nyberg et al., 2017; Mandrup et al., 2018; Torres et al., 2018; Greendale et al., 2019; El Khoudary et al., 2020; Juppi et al., 2020; Pellegrino et al., 2022; Kleis-Olsen et al., 2024). MHT should not be assumed universally to be an effect modifier. Its analytical role must be specified *a priori* from the study question and causal model and may be that of an exposure, confounder, mediator, source of staging ambiguity, or hypothesized effect modifier. Interaction terms or stratified estimates by formulation, route, dose, duration, and time since initiation are appropriate when effect modification is specifically hypothesized and adequately powered; otherwise restriction, adjustment, or separate exposure analyses should follow the prespecified causal model.

## 4 Exercise as a metabolic challenge and intervention

### 4.1 The acute exercise lactate component measurement profile

Exercise provides a controlled perturbation that increases ATP demand, glycolytic flux, muscle blood flow, oxygen consumption, and interorgan substrate exchange (Brooks, 1986; Gladden, 2004; Hashimoto and Brooks, 2008; Goodpaster and Sparks, 2017; Hui et al., 2017; Brooks, 2018; Ferguson et al., 2018; Rabinowitz and Enerbäck, 2020). An exercise lactate curve may therefore reveal stage-related differences not apparent from fasting glucose, fasting insulin, or resting lactate (Gladden, 2004; Moreau et al., 2013; Goodpaster and Sparks, 2017; Hui et al., 2017; Brooks, 2018; Ferguson et al., 2018; Rabinowitz and Enerbäck, 2020). Interpretation, however, depends strongly on how exercise intensity and completed work are standardized (Faude et al., 2009; Moreau et al., 2013; San-Millán and Brooks, 2018).

Testing at the same absolute workload estimates the metabolic cost of the same external task, although differences may partly reflect fitness and active muscle mass (Moreau et al., 2013; San-Millán and Brooks, 2018; Juppi et al., 2020). Testing at the same percentage of peak oxygen uptake (VO_2_peak) or peak power reduces some fitness-related differences but can place participants in different physiological intensity domains (Faude et al., 2009; San-Millán and Brooks, 2018). Whenever feasible, studies should pair fixed absolute stages with relative stages anchored to individual gas-exchange thresholds, critical power, or another prespecified domain boundary, and report power, stage duration, oxygen uptake, and completed work. This paired design helps separate task efficiency from relative metabolic strain (Faude et al., 2009; Moreau et al., 2013; San-Millán and Brooks, 2018).

Blood-lactate thresholds and MLSS are established descriptors of endurance physiology, but their definitions and protocols are not interchangeable (Beneke, 2003; Faude et al., 2009). In 75 postmenopausal women, mean MLSS velocity was 7.1 ± 1.0 km·h^−1^; a lactate-threshold-derived predictor explained 80% of its variance, a multivariable model explained 85%, and adding one constant-velocity test increased explained variance to 89.2% (Gil-Rey et al., 2019). These data demonstrate the feasibility and predictive performance of concentration-based exercise testing in postmenopausal women. They do not establish a menopause effect, because the study had no premenopausal or perimenopausal comparator, and they do not identify lactate turnover because Ra, Rd, oxidation, and recycling were not measured (Gil-Rey et al., 2019).

Serial recovery measurements can complement peak concentration, but the post-exercise decline is jointly influenced by distribution, ongoing production, oxidation, gluconeogenesis, blood flow, and active versus passive recovery (Pilegaard et al., 1999; Dubouchaud et al., 2000; Gladden, 2004; Thomas et al., 2005; Maciejewski et al., 2016; Brooks, 2018; Ferguson et al., 2018; Rabinowitz and Enerbäck, 2020). Muscle MCT content and oxidative capacity have been associated with fitted blood-lactate concentration-recovery parameters after supramaximal exercise, but small observational studies cannot identify transport as the sole determinant of Rd or whole-body metabolic clearance (Thomas et al., 2005; Maciejewski et al., 2016). A prespecified component measurement profile should report exercise power, oxygen consumption, serial concentration, peak, time to peak, area under the curve, and fitted concentration-recovery parameters as separate outcomes rather than combine them into an unvalidated index (Gladden, 2004; Thomas et al., 2005; Faude et al., 2009; Moreau et al., 2013; Maciejewski et al., 2016; Brooks, 2018; Ferguson et al., 2018; San-Millán and Brooks, 2018; Gil-Rey et al., 2019; Rabinowitz and Enerbäck, 2020). Sampling should be dense enough to capture a delayed peak and the chosen model (for example, end exercise and 1, 3, 5, 7, 10, 15, 20, and 30 min when appropriate); a fitted slope or rate constant must be labeled an indirect concentration-recovery parameter, not lactate clearance, Rd, or whole-body metabolic clearance.

### 4.2 Training adaptation and exercise modality

Exercise training increases markers of skeletal muscle mitochondrial content and capillarization across endurance, high-intensity interval, and sprint-interval protocols in general adult populations (Mølmen et al., 2025). Endurance and high-intensity training can also increase MCT1, alter MCT4, modify LDH expression, and accelerate aspects of post-exercise lactate handling (Pilegaard et al., 1999; Dubouchaud et al., 2000; Juel et al., 2004; Thomas et al., 2005; Bishop et al., 2008; Summermatter et al., 2013; Benítez-Muñoz et al., 2024). These observations derive from non-perimenopausal or indirect populations and justify testing whether training changes the proposed component measurement profile; they do not establish that these pathways mediate a menopause-specific lactate defect (Pilegaard et al., 1999; Dubouchaud et al., 2000; Juel et al., 2004; Thomas et al., 2005; Bishop et al., 2008; Summermatter et al., 2013; Nyberg et al., 2017; Mandrup et al., 2018; Yoh et al., 2023; Benítez-Muñoz et al., 2024; Mølmen et al., 2025).

A 2026 single-group study in active postmenopausal women reported higher power, oxygen uptake, and blood lactate at the intensity of maximal carbohydrate oxidation after six weeks of high-intensity interval training (Monferrer-Marín et al., 2026). Because it included no reproductive-stage comparator and did not measure Ra, Rd, oxidation, or recycling, it provides indirect evidence of training responsiveness rather than evidence for altered lactate kinetics during natural perimenopause (Monferrer-Marín et al., 2026).

Moderate-intensity continuous training may favor mitochondrial expansion, capillarization, and sustained lactate oxidation at submaximal workloads (Dubouchaud et al., 2000; Juel et al., 2004; Mølmen et al., 2025). High-intensity interval training typically produces larger acute glycolytic and blood-lactate responses and can promote adaptations in mitochondria, vasculature, glucose uptake, buffering capacity, and lactate transport (Pilegaard et al., 1999; Bishop et al., 2008; Nyberg et al., 2017; Mandrup et al., 2018; Mølmen et al., 2025). Resistance training increases muscle mass and glycogen-storage capacity but produces protocol-dependent blood-lactate responses determined by load, repetitions, recruited muscle mass, and interset recovery (Juel et al., 2004). A large acute blood-lactate response is neither necessary nor sufficient to demonstrate an effective resistance-training stimulus (Gladden, 2004; Juel et al., 2004; Brooks, 2018; Ferguson et al., 2018; Rabinowitz and Enerbäck, 2020).

Existing research does not identify a single best exercise modality for lactate handling during perimenopause (Bishop et al., 2008; Faude et al., 2009; Nyberg et al., 2017; Mandrup et al., 2018; San-Millán and Brooks, 2018; Gil-Rey et al., 2019; Benítez-Muñoz et al., 2024; Mølmen et al., 2025). Comparing protocols only by the lowest post-exercise concentration would be physiologically misleading because a potent training stimulus may generate substantial lactate while improving later transport or oxidation (Pilegaard et al., 1999; Dubouchaud et al., 2000; Gladden, 2004; Juel et al., 2004; Thomas et al., 2005; Summermatter et al., 2013; Brooks, 2018; Ferguson et al., 2018; Rabinowitz and Enerbäck, 2020; Benítez-Muñoz et al., 2024). Combined aerobic and resistance training remains a reasonable clinical strategy for oxidative capacity and muscle mass, but its effect on lactate flux across menopausal stages is untested (Pilegaard et al., 1999; Dubouchaud et al., 2000; Juel et al., 2004; Thomas et al., 2005; Summermatter et al., 2013; Nyberg et al., 2017; Mandrup et al., 2018; Juppi et al., 2020; Benítez-Muñoz et al., 2024; Mølmen et al., 2025).

## 5 An exercise-challenge lactate framework for future research

The primary menopause-specific hypothesis is that reproductive stage or ovarian-hormone variability may alter coordination between systemic lactate Ra and Rd, or the variability of those responses, rather than cause a consistent shift in one blood-lactate value (Brooks, 1986; Gladden, 2004; Harlow et al., 2012; Goodpaster and Sparks, 2017; Hui et al., 2017; Brooks, 2018; Ferguson et al., 2018; Rabinowitz and Enerbäck, 2020). This hypothesis extends rather than duplicates established shuttle, MLSS, metabolic-flexibility, and Lactate-Carbohydrate models (Brooks, 1986; Beneke, 2003; Faude et al., 2009; Brooks, 2018; San-Millán and Brooks, 2018; Alkhatib, 2022). A stage-related difference should therefore be tested across multiple standardized workloads and recovery, with direct tracer outcomes whenever feasible. Training-related change could appear as higher sustainable power at a given concentration, a different serial concentration-recovery curve, or greater tracer-measured oxidation, but each outcome must remain separate until menopause-specific validity and reliability are established (Pilegaard et al., 1999; Dubouchaud et al., 2000; Juel et al., 2004; Thomas et al., 2005; Bishop et al., 2008; Faude et al., 2009; Moreau et al., 2013; Summermatter et al., 2013; Nyberg et al., 2017; Mandrup et al., 2018; San-Millán and Brooks, 2018; Gil-Rey et al., 2019; Benítez-Muñoz et al., 2024; Mølmen et al., 2025).

Future studies should classify reproductive stage using STRAW + 10 criteria, separating premenopause, early and late perimenopause, early postmenopause, and later postmenopause (Harlow et al., 2012). STRAW + 10 stage should be the primary exposure definition for participants whose natural menstrual history is interpretable; repeated estradiol and FSH samples should characterize hormone variability rather than replace menstrual-history staging (Harlow et al., 2012). Sampling on separate days or visits, with cycle timing, amenorrhea duration, assay platform, and lower limits of quantification reported, is preferable to a single hormone value. Because hormonal contraception and MHT can obscure bleeding patterns and endogenous endocrine criteria, investigators must prespecify a hormone-user policy. For primary natural-stage comparisons, current hormonal-contraception users and participants whose natural stage cannot be reconstructed should be excluded from the stage-defined analysis or analyzed as separate exogenous-hormone strata, not assigned a stage from contemporaneous estradiol or FSH. Participants who began stable MHT after a clinically documented final menstrual period may be classified from the pre-treatment menstrual history, but MHT exposure should be analyzed separately and tested in sensitivity analyses. Initiation, cessation, or formulation change during follow-up should trigger a prespecified censoring, reclassification, or separate-exposure rule. Formulation, route, dose, duration, adherence, indication, and time since initiation or cessation should be recorded for both MHT and hormonal contraception (Harlow et al., 2012; Moreau et al., 2013; Pellegrino et al., 2022; Kleis-Olsen et al., 2024).

Stable-isotope lactate tracers, with an appropriate steady-state or non-steady-state kinetic model, can estimate systemic Ra, Rd, and oxidation; lactate-carbon recycling requires additional tracer or product measurements (Gladden, 2004; Hui et al., 2017; Brooks, 2018; Ferguson et al., 2018; Hui et al., 2020; Rabinowitz and Enerbäck, 2020). Blood concentration alone cannot identify these fluxes or whole-body metabolic clearance. No such tracer study has been reported during natural perimenopause. Muscle biopsies can assess MCT1, MCT4, LDHA, LDHB, PGC-1α, ERRα, ERα, mitochondrial respiration, citrate synthase activity, fiber type, and capillary density (Pilegaard et al., 1999; Dubouchaud et al., 2000; Juel et al., 2004; Thomas et al., 2005; Moreau et al., 2012; Summermatter et al., 2013; Nyberg et al., 2017; Yoh et al., 2023; Benítez-Muñoz et al., 2024; Mølmen et al., 2025). These measurements should be integrated analytically while retaining their distinct biological meanings because circulating lactate reflects multiple organs and processes (Gladden, 2004; Hui et al., 2017; Brooks, 2018; Ferguson et al., 2018; Hui et al., 2020; Rabinowitz and Enerbäck, 2020).

Dietary carbohydrate and total energy intake, prior exercise, muscle glycogen, fasting duration, alcohol and caffeine, hydration, sampling site, analyzer, environmental temperature, and time of day can influence exercise physiology or blood-lactate measurements and should be standardized or recorded (Gladden, 2004; Faude et al., 2009; Brooks, 2018; Ferguson et al., 2018; San-Millán and Brooks, 2018; Rabinowitz and Enerbäck, 2020). Recovery must be prespecified as active or passive, with posture, duration, cadence or workload, ambient conditions, and sampling times held constant. Covariates should be selected using a prespecified causal model rather than indiscriminate adjustment; minimum candidates include age, total and visceral adiposity, active muscle mass, habitual physical activity, cardiorespiratory fitness, insulin sensitivity, smoking, medication use (including glucose-lowering and cardiovascular drugs), exogenous hormone use, and cardiometabolic disease (Janssen et al., 2008; Lovejoy et al., 2008; Abildgaard et al., 2013; Greendale et al., 2019; El Khoudary et al., 2020; Juppi et al., 2020; Kleis-Olsen et al., 2024). The role of MHT must be determined *a priori* for each analysis. When MHT is hypothesized to modify an association, interaction or stratified analyses should be prespecified and powered; when it is a confounder, mediator, or exposure of interest, the corresponding adjustment, mediation, restriction, or separate-exposure strategy should follow the causal model. Fitness, adiposity, and insulin sensitivity should be handled in the same question-specific manner rather than automatically designated effect modifiers. Symptoms such as fatigue, sleep disturbance, and vasomotor symptoms may be related outcomes, but association would not establish lactate as their cause (Harlow et al., 2012; Moreau and Hildreth, 2014; El Khoudary et al., 2020).

Before between-stage inference, investigators should include familiarization and quantify analytical and biological reliability using duplicate baseline tests in all participants or a representative subset. Reports should include analyzer coefficient of variation, within-participant coefficient of variation, intraclass correlation, typical error, and smallest detectable change; repeated visits within a reproductive stage are desirable to separate measurement error from short-term hormone and symptom variability.

Serial blood-lactate data should be analyzed with a prespecified mixed-effects model or justified nonlinear kinetic model, including participant-level random effects and stage-by-intensity or stage-by-time interactions (Gladden, 2004; Thomas et al., 2005; Faude et al., 2009; Brooks, 2018; Ferguson et al., 2018; San-Millán and Brooks, 2018; Rabinowitz and Enerbäck, 2020). Absolute-load and relative-domain tests should be analyzed separately; baseline concentration, completed work, and relevant covariates should be handled explicitly. Peak, time to peak, area under the curve, and concentration-recovery parameters require prespecified windows and confidence intervals, with multiplicity control, missing-data procedures, and sensitivity analyses reported. A fitted concentration-decay parameter must not be interpreted as Rd or whole-body metabolic clearance without tracer data.

Emerging lactate-signaling mechanisms should remain exploratory and outside the core component measurement profile. Histone lactylation is an emerging mechanism through which cellular metabolic state may influence gene regulation, but the foundational evidence was generated outside menopausal exercise physiology (Zhang et al., 2019). No study has tested exercise-related histone lactylation during natural perimenopause, and current evidence does not support a perimenopause-specific benefit, mediator, biomarker, or therapeutic target (Summermatter et al., 2013; Zhang et al., 2019; Yoh et al., 2023). If retained in future studies, histone lactylation should be treated as hypothesis-generating and interpreted separately from lactate concentration and flux.

## 6 Limitations

This is a structured narrative Mini Review, not a registered systematic review. Complete search strings and eligibility rules are reported in **Supplementary Methods S1**, but title/abstract screening, full-text eligibility assessment, evidence classification, and data extraction were performed by one reviewer rather than in duplicate. A formal de-duplication log, flow diagram, risk-of-bias assessment, and quantitative synthesis were not produced; selection and publication bias therefore remain possible. The statement that no direct natural-perimenopause lactate-flux study was identified is limited to the documented databases, date range, citation chaining, and eligibility criteria and should not be interpreted as proof that no relevant evidence can exist. The mechanistic discussion necessarily extrapolates from established postmenopause, animal or cellular models, and other exercise populations. Heterogeneous exercise protocols and concentration-only outcomes further limit comparison. Finally, the lactate metabolic plasticity framework has no validated composite index, cutoff, reliability threshold, or demonstrated clinical utility.

## 7 Conclusion

The established science is clear that lactate is a circulating fuel, carbon shuttle, gluconeogenic precursor, and protocol-dependent marker of exercise metabolism (Brooks, 1986; Beneke, 2003; Gladden, 2004; Faude et al., 2009; Goodpaster and Sparks, 2017; Hui et al., 2017; Brooks, 2018; Ferguson et al., 2018; San-Millán and Brooks, 2018; Rabinowitz and Enerbäck, 2020; Alkhatib, 2022). The unresolved question is specific to reproductive aging: no identified study has quantified how natural perimenopause affects lactate Ra, Rd, oxidation, recycling, or whole-body metabolic clearance. The proposed lactate metabolic plasticity framework addresses that gap by combining STRAW + 10 staging and repeated ovarian-hormone measurements with standardized exercise and recovery, direct tracer fluxes, and muscle or vascular phenotyping. Available quantitative evidence supports relevant stage differences in body composition, substrate use, and endothelial function, and concentration-based MLSS testing is feasible in postmenopausal women, but none of those observations demonstrates altered lactate turnover (Lovejoy et al., 2008; Moreau et al., 2012; Abildgaard et al., 2013; Gil-Rey et al., 2019). The framework therefore remains hypothesis-generating. Future longitudinal studies should report concentration and tracer outcomes separately, compare absolute and relative workloads, and distinguish natural perimenopause from established postmenopause and exogenous-hormone exposure. Until those data exist, the framework must not be used as a validated biomarker, composite index, clinical phenotype, or basis for selecting one exercise modality.

## Supplementary material

Supplementary Methods S1 Search strategy and eligibility criteria for the structured narrative review, including core search strings, supplementary search families, and database-specific field and date-expansion rules.
