# Supplementary material for: Lactate metabolic plasticity across the menopausal transition: a hypothesis-generating framework linking ovarian hormone variability, exercise, and skeletal muscle remodeling
Source: Front Physiol. 2026 Oct 28;17:1954454. doi: 10.3389/fphys.2026.1954454 (PMC13752543; doi:10.3389/fphys.2026.1954454)
Supplement: Supplementary file 1 [file SupplementaryFile1.docx]

**Supplementary Methods S1**

# S1.1 Core database searches

## PubMed/MEDLINE core search

((menopaus*[Title/Abstract] OR perimenopaus*[Title/Abstract] OR "menopausal transition"[Title/Abstract] OR postmenopaus*[Title/Abstract]) AND (lactate[Title/Abstract] OR "lactate kinetics"[Title/Abstract] OR "lactate turnover"[Title/Abstract] OR "rate of appearance"[Title/Abstract] OR "rate of disappearance"[Title/Abstract] OR oxidation[Title/Abstract] OR clearance[Title/Abstract] OR MCT1[Title/Abstract] OR MCT4[Title/Abstract] OR LDHA[Title/Abstract] OR LDHB[Title/Abstract]) AND (exercise[Title/Abstract] OR training[Title/Abstract] OR "skeletal muscle"[Title/Abstract])) AND ("1900/01/01"[Date - Publication] : "2026/08/30"[Date - Publication])

## Europe PMC core search

((TITLE_ABS:menopaus* OR TITLE_ABS:perimenopaus* OR TITLE_ABS:"menopausal transition" OR TITLE_ABS:postmenopaus*) AND (TITLE_ABS:lactate OR TITLE_ABS:"lactate kinetics" OR TITLE_ABS:"lactate turnover" OR TITLE_ABS:"rate of appearance" OR TITLE_ABS:"rate of disappearance" OR TITLE_ABS:oxidation OR TITLE_ABS:clearance OR TITLE_ABS:MCT1 OR TITLE_ABS:MCT4 OR TITLE_ABS:LDHA OR TITLE_ABS:LDHB) AND (TITLE_ABS:exercise OR TITLE_ABS:training OR TITLE_ABS:"skeletal muscle")) AND FIRST_PDATE:[1900-01-01 TO 2026-08-30]

# S1.2 Supplementary mechanistic searches

**Platform expansion rule.** Each search family below was run in both databases. In PubMed, every unquoted free-text term was searched in [Title/Abstract] and the publication-date limit shown above was applied. In Europe PMC, the same term was searched with TITLE_ABS: and the FIRST_PDATE limit shown above was applied. The full concept strings are listed below so the expansion is reproducible.

## Supplementary search A: Perfusion and vascular function

(menopaus* OR perimenopaus* OR "menopausal transition" OR postmenopaus*) AND (estradiol OR estrogen OR "ovarian hormone*" OR "menopausal hormone therapy" OR "hormone replacement therapy") AND (perfusion OR "blood flow" OR endothelial OR vascular OR capillary OR capillarization) AND (exercise OR training OR "skeletal muscle")

## Supplementary search B: Mitochondrial and substrate metabolism

(menopaus* OR perimenopaus* OR "menopausal transition" OR postmenopaus*) AND (estradiol OR estrogen OR "ovarian hormone*" OR "menopausal hormone therapy" OR "hormone replacement therapy") AND (mitochondri* OR respiration OR "oxidative capacity" OR "substrate oxidation" OR "fat oxidation" OR "insulin sensitivity" OR "metabolic flexibility") AND (exercise OR training OR "skeletal muscle")

## Supplementary search C: Candidate transport and regulatory pathways

(menopaus* OR perimenopaus* OR "menopausal transition" OR postmenopaus*) AND (estradiol OR estrogen OR "ovarian hormone*" OR "menopausal hormone therapy" OR "hormone replacement therapy") AND ("PGC-1α" OR "PGC-1alpha" OR PGC1A OR "ERRα" OR "ERR-alpha" OR ESRRA OR MCT1 OR MCT4 OR SLC16A1 OR SLC16A3 OR LDHA OR LDHB) AND (exercise OR training OR "skeletal muscle")

## Supplementary search D: Lactate signaling and lactylation

(menopaus* OR perimenopaus* OR "menopausal transition" OR postmenopaus*) AND (lactate OR lactylation OR "histone lactylation") AND (exercise OR training OR muscle OR metabolism)

# S1.3 Citation tracking and bibliographic verification

**Backward citation tracking.** Reference lists of included original studies, relevant reviews, and staging guidance were inspected for additional eligible or contextual records.

**Forward citation tracking.** Available cited-by links associated with key reviews and original studies in Europe PMC, PubMed-linked publisher records, and publisher pages were screened through 30 August 2026.

**Metadata verification.** Crossref and publisher or PubMed records were used to verify author lists, titles, journals, years, pagination or article numbers, and DOI information. Metadata searches were not treated as independent evidence searches.

# S1.4 Screening and eligibility process

One reviewer screened titles and abstracts. Full text was obtained when a record involved a relevant reproductive stage, lactate concentration or flux, exercise or skeletal muscle, or a mechanism prespecified in the evidence hierarchy.

Direct natural-perimenopause evidence required a human cohort undergoing the natural menopausal transition and an identifiable reproductive stage based on menstrual history, STRAW+10-compatible criteria, or an equivalent reported definition.

For the central evidence-gap claim, an eligible study also had to measure lactate Ra, Rd, oxidation, carbon recycling, or model-derived whole-body metabolic clearance at rest, during exercise, or during standardized recovery. Blood lactate concentration alone did not satisfy this flux criterion.

Records were excluded from the direct natural-perimenopause category when they involved only established postmenopause, surgical or treatment-induced menopause, an unidentifiable stage, no eligible lactate outcome, or a disease-specific cohort in which disease and menopause effects could not be separated.

Postmenopausal human studies, other adult studies, animal models, and cell studies were retained only as indirect evidence or biological plausibility. Staging guidance and reviews were used for definitions, context, and citation chaining.

Evidence was classified as direct natural-perimenopause human evidence; indirect postmenopausal or other-human evidence; animal or cellular evidence; or established general lactate physiology.

No eligible study met all criteria for direct measurement of lactate Ra, Rd, oxidation, recycling, or whole-body metabolic clearance during natural perimenopause. This result is limited to the databases, search date, citation tracking, and eligibility rules reported here.

# S1.5 Methodological limitations of the search

This was a structured narrative search rather than a registered systematic review. Screening, evidence classification, and extraction were not performed in duplicate. A formal de-duplication log, record-count flow diagram, risk-of-bias assessment, and quantitative synthesis were not produced. The complete strings and decision rules above improve reproducibility and define the scope of the negative finding, but they do not eliminate selection or publication bias.
